# The determinants of premature birth during the year 2018 at the General Reference Hospital of Malemba in the Democratic Republic of Congo

**DOI:** 10.11604/pamj.2020.37.30.25205

**Published:** 2020-09-08

**Authors:** Fiston Ilunga Mbayo, Yannick Bienge Nsenga, Gloire Kasongo Lupitshi, Kennedy Twite Nyemba, Cédrick Mwamba Mpingisha, Justin Batambe Kambala, Gaetan Kabongo Muzinga

**Affiliations:** 1 Malemba General Reference Hospital, Malemba, Democratic Republic of Congo,; 2 University of Malemba Nkulu, Malemba, Democratic Republic of Congo

**Keywords:** Premature delivery, determinants, Malemba

## Abstract

Premature birth remains a major concern for obstetric and pediatric teams. The objective of our work is to determine the causes and frequencies of premature childbirth in our environment, more particularly at the HGR Malemba. This is a descriptive cross-sectional study conducted in the obstetrics and gynecology department of the HGR Malemba. Who included 24 premature deliveries between 1^st^ January and 31^st^ December 2018. The hospital frequency of preterm birth was 4.6%, the average age of childbirth was 28 ± 6.7 years, married births were 75%, housewives were 45.8%, the women born were large multiparous in 41.7%, the pathologies occurring during pregnancy: urinary tract infections (75%); malaria (25%), maternal age <18 years or >35 years and urinary tract infections constitute important risk factors for preterm birth. The study we conducted shows that the rate of premature delivery remains high in the city of Malemba. The early identification of the cause allows their reduction through the improvement of the health care system in our environment.

## Introduction

Preventing premature childbirth is at the forefront of obstetrics concerns, so uncertain the future of premature babies. This prematurity is a major risk factor for morbidity and mortality neonatal tale. The frequency of premature childbirth is high, variable depending on the place and time. It depends above all on the prevention and treatment of the threat of preterm birth. The Democratic Republic of Congo is one of the 10 countries in the world with the highest rates of premature births with 341,400 premature babies each year and half of premature babies born in 32 weeks of amenorrhea die [1]. A number of extremely varied risk factors contribute to the occurrence of preterm birth. This is why, as a scrutineer and out of scientific curiosity, we have chosen to enter into the intricacies of premature childbirth in order to seize the ins and outs.

**General objective:** study the determinants of premature delivery in our study environment.

**Specific objectives:** determine the frequency of premature deliveries at the Malemba General Referral Hospital; determine the factors influencing premature deliveries within the said hospital.

## Methods

**Study framework:** our study was carried out in the maternity unit of the obstetric gynecology service of the Malemba General Referral Hospital located in the Haut Lomami Province in the Democratic Republic of Congo.

### Type and period of study

**Study type:** this is a retro-prospective cross-sectional descriptive study on premature deliveries in the obstetric gynecology department of Malemba Hospital.

**Study period:** our study took place over a period from 1^st^ January to 31^st^ December 2018, 12 months.

**Population and sampling:** pregnant women admitted or referred for preterm delivery in the obstetric gynecology department of the HGR Malemba during the period of our research.

### Sampling

**Inclusion criteria:** pregnant women received in the delivery room at a term between 22 to 37 weeks; parturientes presenting a complete file.

**Exclusion criteria:** pregnant women who gave birth before 22 weeks old and after 37 weeks old, parturients with an incomplete file, pregnant women who gave birth out of service.

**Data management and collection:** data were collected from partograms, hospitalization records and the HGR Malemba delivery register during our study period. At the end of this collection, a sample of 24 premature deliveries was selected.

**Ethical considerations:** the anonymity of all the women born was respected, the consent was obtained by the management of the Hospital of Malemba and the department of obstetric gynecology.

## Results

**Frequency:** during our study we recorded 24 premature deliveries from 1^st^January to 31^st^December 2018, a total of 517 deliveries a frequency of 4.6%.

**Age:** it emerges from this table (Table 1) that 17 delivered or 70.8% were between 18 and 35 years old against 19 delivered or 8.4% were under 18 years old. The mean age was 28.7 ± 6.7 years with the extremes being 15 and 46 years.

**Table 1:** distribution of women giving birth by age

Age groups (in years)	Effective	Percentage
<18	2	8.4
18 to 35	17	70.8
>35	5	20.8
Total	24	100.00

**Marital status:** analysis of this table (Table 2) shows that 18 delivered, i.e. 75% were married against 1 childbirth, or 4.1% was widowed; single and divorced women represented 12.5% and 4.2% respectively.

**Table 2:** distribution of women giving birth by marital status

Marital status	Effective	Percentage
Single	3	12.5
Divorcee	2	8.3
Married	18	75
Widow	1	4.2
Total	24	100.00

**Profession:** it emerges from this table (Table 3) that 45.8% of our deliveries were housewives with a number of 11 deliveries against a childbirth who was a civil servant, i.e. 4.2%; 10 women delivered were liberal or 41.7% and 2 women delivered were pupils or students or 8.3%.

**Table 3:** breakdown of women giving birth by profession

Profession	Effective	Percentage
Pupil or student	2	8.3
State worker	1	4.2
Liberal	10	41.7
Household	11	45.8
Total	24	100.00

**Parity:** it emerges from this table (Table 4) that 10 delivered or 41.7% were large multiparous against 2 delivered or 8.3% were pauciparous; 8 delivered or 33.3% were multiparous; and first-time mothers were 4, i.e. 16.7%.

**Table 4:** distribution of women giving birth according to parity

Parity	Effective	Percentage
Large multipare	10	41.7
Multiparous	8	33.3
Pauciparous	2	8.3
Primiparous	4	16.7
Total	24	100.00

**Gesture:** this table (Table 5) shows that 9 delivered or 37.5% were large multigestants against 4 women delivered or 16.7% were paucigestous; the multigestes and the primigestes were respectively 6(25%) and 5(20.8%).

**Table 5:** breakdown of women giving birth according to pregnancy

Gesture	Effective	Percentage
Primigest (1)	5	20.8
Paucigest (2-3)	4	16.7
Multigest (4-6)	6	25
Large multigest (≥7)	9	37.5
Total	24	100.00

**Pathologies during pregnancy:** analysis of this table (Table 6) shows that urinary tract infection represented the most frequent pathology during pregnancy with 75%, i.e. 18 women delivered against one childbirth or 4.2% had diabetes; malaria and hypertension accounted for 25%, or 6 deliveries and 8.3%, or 2 deliveries, respectively.

**Table 6:** distribution of women giving birth according to the pathologies occurring during the current pregnancy

Pathologies during pregnancy	Effective	Percentage
Urinary tract infection	18	75
HTA	2	8.3
Malaria	6	25
Diabetes	1	4.2

**Type of pregnancy:** this table (Table 7) shows that 20 childbirths or 83.3% had a single fetal pregnancy against one childbirth or 4.2% had a multiple pregnancy, 3 women born or 12.5% had a twin pregnancy.

**Table 7:** breakdown of women giving birth by type of pregnancy

Type of pregnancy	Effective	Percentage
Twin pregnancy	3	12.5
Mono-fetal pregnancy	20	83.3
Triple pregnancy	1	4.2
Total	24	100.00

## Discussion

**Frequency:** our study shows that out of 517 pregnant women who gave birth at the Malemba General Hospital in DR Congo during the period from January to December 2018 (12 months), we recorded 24 premature deliveries, i.e. a frequency of 4.6%. This frequency is explained by factors related to parturients, medical factors and socio-economic factors. Confronted with data from the literature, our frequency is higher than that of Sanogo [2] with 1.9% and that of Traoré [3] with 1.26% and is proportional to that of Pambou at the Brazzaville CHU [4] which found 4.7% as a frequency. Our increase in the premature birth rate could be explained by the late and inappropriate management of PAD by the department, resulting in premature birth; ignorance of parturients; lack of prenatal care; the unfavorable socio-economic conditions of women giving birth.

**Age of childbirth:** the maternal age group the most exposed during our study corresponds well to that of the woman in full genital activity in 70.8%, i.e. 17 women born. But premature childbirth was also found in adolescents and elderly women with 8.4% and 20.8% respectively. Contrary to a study carried out in France, the age group most represented for premature birth is that of ≤18 years and that of over 40 years [5] and after (Ancel, 2002) [6], these are underage women over 35 years of age who are at risk of PAD and therefore susceptible to premature delivery. At the Brazzaville University Hospital (Pambou, 2006) [4], the maternal age group most exposed is that of 14-20 years old in 27% of cases, those over 35 representing only 9%. These discrepancies noted by these authors in relation to our results could be explained by the recruitment criteria used. In our traditions, women are only valued by the number of children they have, hence their procreation from adolescence to old age (46 years in our study) Traoré [3].

**Profession:** in our study, 45.8% of the women born were housewives, 41.7% of the women born had a liberal profession. The rest of the population was made up of pupils and students with 8.3% and civil servants with 4.2%. Our results are close to those of Traore [3] who found 45.89% and those of Sanogo [2] with 47.3% for housewives in the general population. This could be explained by the predominance of housewives in the general population in our series.

**Marital status:** in relation to marital status, our study reveals that it is the married births who are more represented 75% followed by single women 12.5%. This rate is consistent with the work already carried out by Traoré [3]. But unlike the study carried out by Mvondo Nicole who found 50.5% of single births [7]. This could be explained by the fact that women, living alone, are often not able to meet all their needs because of low income, compared to women living in a conjugal relationship or not with a man whose psychological support in addition to financial is a strong contributor to monitoring the pregnancy. Other authors had found that single women were more likely to have premature babies. According to El-Sayed, in the United States, the risk of prematurity was high among married women while this risk decreased among unmarried women. But after adjusting certain parameters, marriage had become less protective against prematurity [8].

**Parity and gesture:** parity would not miss a role in the occurrence of premature childbirth either, it reveals that 41.7% of the women born were large multiparas, 33.3% of multiparas, 16.7% of first-time mothers and 8.3%. Pauciparas, although according to Papiernick in France [9], the risk coefficient of preterm birth had a low positive predictive value of 8% in nulliparas and 33% in multiparas, respectively. The most plausible explanation would be cervical strain. Contrary to the study carried out by Mvondo in Yaoundé, primiparity increased the risk of premature childbirth in an increased manner [7]. Amri had found 26.2% of cases in first-time mothers and 25.4% of cases in multipara (≤4 pregnancies) [10]. Large multigestes with 37.5% of cases were the strongest population in this series.

**Associated pathologies:** analysis of our results showed that 75% of our deliveries had a urinary tract infection which led to premature rupture of the membrane. Our results are far superior to that of Sanogo [2] in Bamako in commune V with 27.3%. However, malaria and hypertension play a non-negligible role in the occurrence of premature childbirth, 54.4% of cases of urinary infection were found by Sidibé [11]. In Morocco, infection is incriminated as the most frequent factor of premature delivery with 40.2% of cases including 15.8% urinary tract infections, 14.1% for genital infection and 3.8% for chorioamnionitis [12]. The risk of preterm birth is ten times higher for multiple pregnancies than for single pregnancies. The explanations are mostly uterine distension and premature rupture of membranes more common for years the multiple pregnancy [11,13].

## Conclusion

We carried out a cross-sectional descriptive study on the causes and frequency of premature deliveries, the objectives of which were to determine the frequency and causes of premature deliveries at the Malemba General Referral Hospital: the incidence of premature delivery was 4.6%; the maternal factors that influenced preterm delivery in our study were: urinary tract infections, parity >4, certain pathologies during pregnancy such as malaria; the fetal factors that influenced preterm birth were twin and multiple pregnancies; the early and effective management of PAD would help reduce the rate of premature birth which has serious consequences for both the premature and the mother. Its reduction can only result from the simultaneous combined improvement of health education objectified by the attendance of women in ANC and of the quality of care at the level of health centers.

### What is known about this topic

- Clinical epidemiological profile of preterm deliveries.

### What this study adds

- Determine the determinants of premature deliveries at the Malemba General Referral Hospital;
- Determine the factors influencing premature deliveries within the said hospital.
